# Differences in trunk and lower extremity muscle activity during squatting exercise with and without hammer swing

**DOI:** 10.1038/s41598-022-17653-7

**Published:** 2022-08-04

**Authors:** Koji Murofushi, Tomoki Oshikawa, Koji Kaneoka, Hiroshi Akuzawa, Daisuke Yamaguchi, Sho Mitomo, Hidetaka Furuya, Kenji Hirohata, Kazuyoshi Yagishita

**Affiliations:** 1 Sports Science Center, Tokyo Medical and Dental University (TMDU), 1-5-45 Yushima, Bunkyo-ku, Tokyo, Zip code 113-8510 Japan; 2 Japan Sports Agency, Tokyo, Japan; 3 Faculty of Sport Science, Waseda University, Tokyo, Japan; 4 Niigata University of Health and Welfare, Niigata, Japan; 5 Clinical Center for Sports Medicine and Sports Dentistry, Tokyo Medical and Dental University, Tokyo, Japan; 6 Department of Rehabilitation, Sonoda Third Hospital/Tokyo Medical Institute Tokyo Spine Center, Tokyo, Japan

**Keywords:** Health care, Public health

## Abstract

Perturbation exercises enhance lower limb and trunk muscles, and adding swing perturbation while loading during exercise might improve muscle activation or strength. This study aimed to check variations in trunk and lower limb muscle activity during conventional isometric squats, and whether it will change with or without swing using the Hammerobics-synchronized squat method. Twelve healthy men participated in this study. Activities for the abductor hallucis, tibialis anterior, tibialis posterior, peroneus longus, rectus femoris, biceps femoris long head, semitendinosus, gluteus maximus, multifidus, and internal oblique muscles were measured using surface electromyography during a Hammerobics-synchronized squat and conventional isometric squat. Muscle activities were statistically compared between squat methods. Hammerobics-synchronized squats significantly activated the abductor hallucis, tibialis anterior, tibialis posterior, peroneus longus, semitendinosus, and multifidus muscles, in both phases, compared with the conventional isometric squats. The Hammerobics-synchronized squat exercise can be considered for trunk and foot stability exercise.

## Introduction

Motor control, which relies on constant communication between the motor and sensory systems, is critical for spinal posture, stability, and movement^1^. Training that involves the use of unstable surfaces has been shown to increase trunk muscle activity and potentially improve trunk stability and balance^2^. Neuromuscular control plays an essential role in trunk stability and lower back pain (LBP) prevention and rehabilitation^3^.

Squat exercises are one of the typical exercises to increase neuromuscular control. The squat exercise is the most well-known and regularly used exercise to activate thighs and numerous muscle groups, not only limited to sports activity but also for rehabilitation to strengthen lower-body muscles and connective tissue after joint injury^4^. As an intervention study, the exercise program with high loads, performed twice per week for eight weeks, demonstrated that leg strength and power improved for young athletes with half back squat exercises^5^. It was shown that power produced during squatting is related to the amount of muscle activity in the lower extremities^6^. In squat exercises, it is important to analyze exercises that can maximize the activity of the lower trunk muscles.

It has been reported that adding varying surfaces and conditions to squat exercises increases muscle activity in the lower extremities and trunk. Schäfer et al. examined unstable situations using internal and external perturbation experimental tests for rowers. Sport-specific movements and postures were performed precisely in the squat and rowing positions using an unstable surface, water-filled pipe, or pushing from a third party^7^. Perturbation-based intervention with 224 exercises (using soft pads, balance cushions, BOSU balls, inverted BOSU balls, Swiss balls, slashpipes, and sling trainers) for a year in adolescent athletes increased trunk muscle strength, reduced strength imbalances between the flexor and extensor muscles, and decreased low-back pain intensity^8^. Furthermore, utilizing unstable situations in exercises can enhance lower limb and trunk muscle stability^9^. Using such water-filled equipment or sandbags enhances lower limb and trunk stability muscles during squatting or clean and jerk exercises for the stabilizing task^10^. Several studies have examined muscle activity according to the swinging perturbation movement in exercise to determine whether muscles can activate effectively. Saeterbakken et al. compared a regular bench press by adding pendulums that swing forward/backwards^11^. Van Gelder et al. examined single-or double-arm kettlebell swinging exercises in the anteroposterior direction to determine whether muscle activation can be increased in the gluteus maximus, gluteus medius, and biceps femoris muscles^12^. Kettlebell swing exercises improve both maximum and explosive strength in a six-week program^13^. Based on these points, adding swing perturbation load when exercising may increase muscle activation or strength.

Hammerobics™ exercises are perturbation exercises that require postural stability and muscle coactivation^14^. The exercise is derived from the athletic event of hammer throwing, which utilizes the concept of parametric excitation. The theoretical system of parametric excitation can be understood by considering a hula-hoop model that uses an energy-pumping system^15,16^. Hammerobics-synchronized squat (HSS) is an exercise that uses swinging perturbation movement in the anteroposterior direction during isometric squats, with competition-approved hammers attached to each side of the Olympic lifting bar while maintaining posture statically^14^. Exercises that involve consciously swinging the hammer while holding the back squat position have the potential to increase the activity of lower extremity and trunk muscles including the foot. However, no such study has examined the activation of the trunk and lower limb muscles during perturbation-based stabilization training in HSS.

The purpose of this study was to compare the muscle activity of the lower trunk muscle groups during conventional squatting and squatting with the addition of hammer swing. For the main task of the HSS, voluntary effort was made in response to the externally applied mechanical loading (hammers movement) while maintaining the swing motion. We hypothesized that the abductor hallucis (Abd H), tibialis anterior (TA), tibialis posterior (TP), and peroneus longus (PL) muscles will be activated in the HSS and that trunk muscles, including the Mul muscle, will demonstrate higher activation with the HSS than with the conventional isometric squat (CIS).

## Methods

### Participants

Twelve healthy men, aged 19–45 years, participated in this study. All the participants were physically active and engaged in at least three practices per week. Before the experiment started, all participants who had severe injuries in the last 3 months or pain on the day of examination were eliminated. The participants were instructed to stop when they felt pain during any of the test phases. None of the participants were interrupted due to injury or discomfort during the examination. This laboratory study used a within-participant repeated-measures design. Muscle activity was the dependent variable, and the form of exercise was the independent variable. The study was approved by the Research Ethics Committee of Tokyo Medical and Dental University (research protocol identification number: M2018-162) and followed the principles of the Declaration of Helsinki (52nd World Medical Association General Assembly Edinburgh, Scotland, October 2000) for medical research involving human subjects. All participants provided written informed consent to study participation.

### Protocol

**Figure 1:**
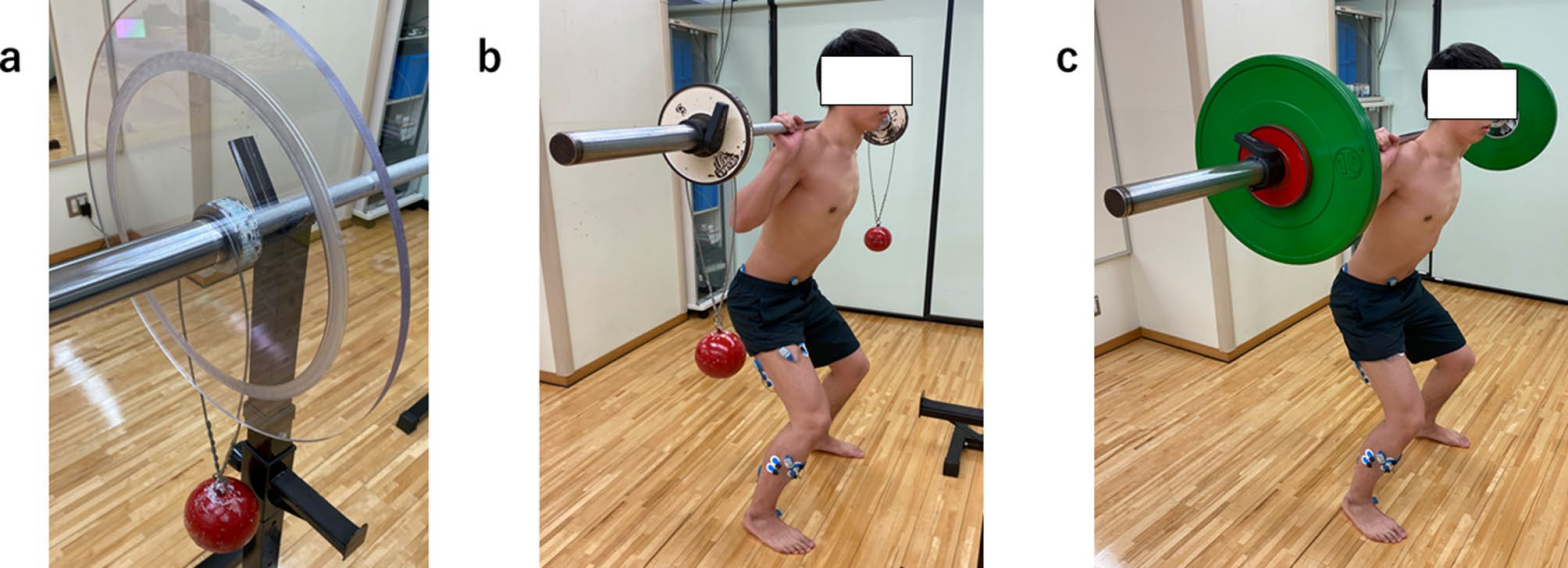
Explanation of Hammerobics-synchronized squat and the conventional isometric squat. (**a**) Hammerobics hammer setup. (**b**) Hammerobics-synchronized squat. (**c**) Conventional isometric squat.

This study measured electromyography (EMG) levels in the lower limb and trunk during two exercises. The dominant leg, defined as the leg that kicks the ball, was used as the measurement leg. The exercise tasks were the HSS and the CIS (Fig. 1). During the exercise, the EMG levels of the Abd H, TA, TP, PL, rectus femoris (RF), biceps femoris long head (BFLH), semitendinosus (ST), gluteus maximus (GM), Mul, and internal oblique muscles (IO) were measured using surface EMG. All exercises were explained verbally and measurements were taken after sufficient practice to familiarize the subjects with the test. All measurements were taken on the same day. Four members of the research team collected the data and uploaded it to a digital platform.

### Data collection and reduction

#### Equipment set-up

**Figure 2:**
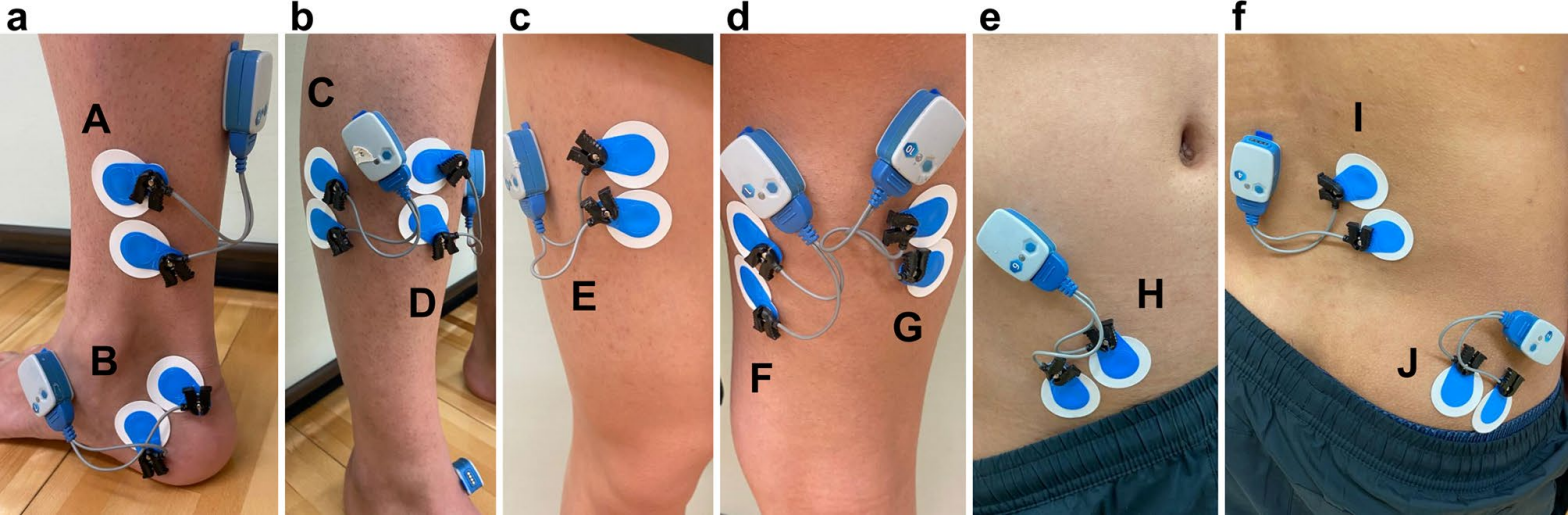
The electrode application site for electromyography. (**a**) medial view of the lower leg, (**b**) anterolateral view of the lower leg, (**c**) anterior view of the upper leg, (**d**) posterior view of the upper leg, (**e**) anterior view of the abdomen, (**f**) posterior view of the lower back. A: Tibialis posterior, B: Abductor hallucis, C: Peroneus longus, D: Tibialis anterior, E: Rectus femoris, F: Semitendinosus, G: Biceps femoris long head, H: Internal oblique, I: Multifidus, J: Gluteus maximus.

EMG signals were recorded during the exercise task using surface EMG (Ultium EMG, EM-U810M8, Tele Myo2400, Noraxon USA Inc., Scottsdale, AZ, USA) and recorded at 2000 Hz with band-pass filtering (10–500 Hz) on a personal computer (EM-P5, Noraxon) using a receiver (EM-U880, Noraxon). The EMG system and the Noraxon Myvideo sytem using a NiNOX 125 were synchronized. Prior to attaching electrodes, the skin was shaved, abraded, and cleaned with alcohol. The electrode application site for EMG was determined according to previous studies^17–19^ and guidelines by SENIAM (URL: http://www.seniam.org/). Surface electrodes (Ambu, Blue Sensor M-00-S, Ballerup, Denmark) were attached 35 mm apart to the Abd H, TA, TP, PL, RF, BFLH, ST, GM, Mul, and IO on the right side (Fig. 2). The electrodes for each muscle were attached parallel to the muscle fibers. The skin impedance was confirmed to be < 5 kΩ before each measurement^20^.

#### Exercise set-up

For the setup, in HSS, a 7.26-kg hammer (φ: 116.5 mm; NISHI Athletics Goods Co. Ltd., Tokyo, Japan) was attached to each end of the Olympic lifting bar by looping the hammer wire. The total length of the equipment from the bottom of the ball to the wire was 0.5 m. Weight set up is described in Table 1. The weight of the equipment was set according to the participant’s body weight range. In CIS, the total weight of the barbell and Olympic lifting bar was adjusted to be equal to the weight of each participant’s HSS. The participants were instructed to perform HSS and CIS exercises under the same conditions. Before starting the task, the participants had the chance to experience both HSS and CIS exercises for 5 to 10 min to familiarize themselves to ensure that the body would be kept at the same height and knee for 90° between exercises. Further, the posture was always visually evaluated by the examiner (knee angle of 90° and consistently using a goniometer). Each exercise type was performed in two trials.

**Table 1:** Weight setup.

Weight range (kg)	Barbell weight
≤ 110	Shaft (20 kg) + 2 hammers (7.26 kg each) + 2 weights (12.5 kg each) = 59.5 kg
95–109	Shaft (20 kg) + 2 hammers (7.26 kg each) + 2 weights (10 kg each) = 54.5 kg
80–94	Shaft (20 kg) + 2 hammers (7.26 kg each) + 2 weights (7.5 kg each) = 49.5 kg
65–79	Shaft (20 kg) + 2 hammers (7.26 kg each) + 2 weights (5 kg each) = 44.5 kg
≤ 64	Shaft (20 kg) + 2 hammers (7.26 kg each) + 2 weights (2.5 kg each) = 39.5 kg

#### Hammerobics synchronized squat

The Hammerobics Synchronized Squat (HSS) is a type of isometric squat exercise in which the two hammers are swung simultaneously in the same direction. This exercise creates anteroposterior and vertical movements by swinging hammers that are hung with wires at each end of an Olympic lifting bar (Fig. 1). When performing, the amplitude of the oscillated hammers was maintained within 90° of the vertical plane for safety^14^. To perform HSS, maintaining an isometric squat position with an upright upper body posture is necessary while moving the hammers steadily in the anteroposterior direction. It should be noted that focus of the exercise is not to see how much amplitude can be applied to the swinging of the hammers, but to maintain the amplitude of swing without disrupting the rhythm of the hammers, using minimal body motion, postural change, and rhythm. During the HSS trial, ten swings were recorded. Hammer movements during the HSS trial were captured by a high-speed camera synchronized with an EMG system.

#### Conventional isometric squat

The Conventional Isometric Squat (CIS) is an isometric exercise using a barbell with the same weight as HSS, in which the individual remains in the squat position to keep the hip and knee angles relatively flexed. The data was recorded for 10 s during the CIS trial. For the data during the HSS trial, three swings of data were extracted from the obtained data and used for the analysis. The movement during the HSS trial was divided into two phases based on the hammer movement captured by the high-speed camera. We defined hammer movement with HSS as front-to-back (FB) and back-to-front (BF). During the FB phase, the hammer moved from the front side of the participants after reaching the highest point, and then to the back side at the highest point. For the BF, the movement of the hammer is the opposite of that in the FB phase, from the back side at the highest to the front side at the highest. The EMG levels in each phase were used for the analysis. In each CIS trial, we recorded 10 s when the participant was in the initial squat posture. Between data on 4.01–7.00 s were used.

All raw EMG signals were rectified and smoothed using a root-mean-square algorithm with a 50-ms time reference. This experimental test was not used for the comparison of muscle activity levels between the muscles. An amplitude comparison of the signals from a given muscle was conducted between the two exercise tasks within an individual in the same session, strictly under the same experimental conditions, and without altering the EMG electrodes^21,22^. The average value used for analysis (μV-s) was calculated and averaged over the three complete swings during the exercise task, and the mean values were used for analysis^23^.

### Data analysis

Data analysis were performed using IBM SPSS Statistics version 25.0 (IBM Corp., Armonk, NY, USA). The Shapiro–Wilk test was performed to confirm normality. Depending on the normality of the distribution, a one-way analysis of variance or the Kruskal–Wallis test was used to examine the difference between the exercise tasks. The post hoc test for one-way analysis of variance or Kruskal–Wallis test was the Bonferroni correction. A p*-*value < 0.05 was considered significant in an a priori power analysis. Data are expressed as the median (interquartile range).

## Results

**Figure 3:**
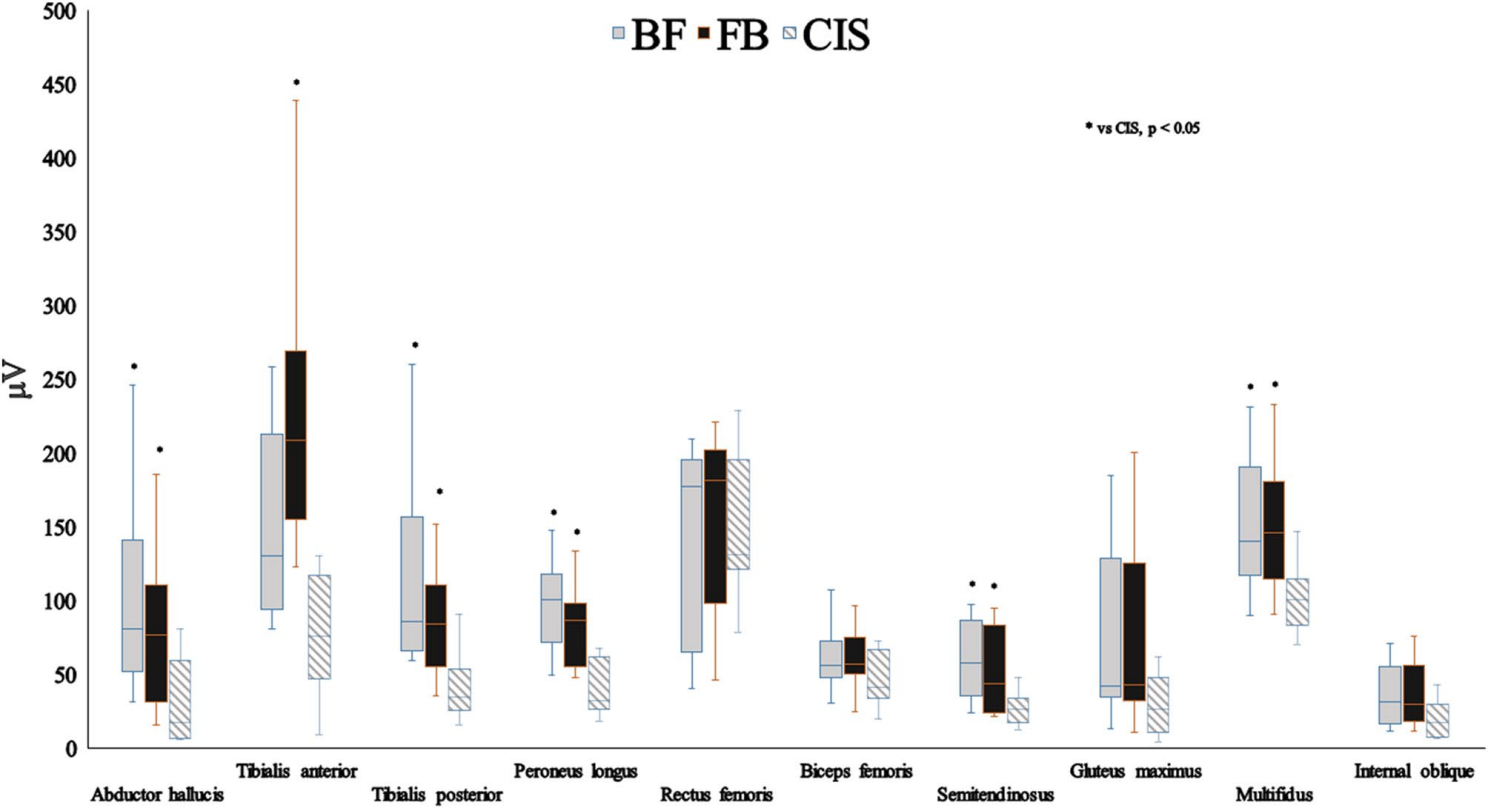
The median and interquartile range of muscle activity during each exercise task. *BF* back-to-front, *FB* front-to-back, *CIS* conventional isometric squat.

For each exercise condition, there was no normality in the muscle activity. Therefore, a non-parametric method was chosen for comparisons between conditions. In the FB phase, Abd H, TA, TP, PL, ST and Mul muscle activity during HSS were significantly higher than those during CIS (Tables 2,3, Fig. 3). In the BF phase, Abd H, TP, PL, ST, and Mul muscle activities in the HSS were significantly higher than those in the CIS (Table 2, Fig. 3).

**Table 2:** The activity of each muscle in BF, FB and CIS.

Muscles	BF median (interquartile range)	FB median (interquartile range)	CIS median (interquartile range)
Abductor hallucis	80.55 (59.31)	77.08 (60.93)	17.1 (38.59)
Tibialis anterior	130.5 (88.05)	208.5 (100.11)	75.87 (63.58)
Tibialis posterior	85.6 (76.61)	84.18 (45.41)	34.85 (26.22)
Peroneus longus	100.9 (38.54)	86.9 (35.9)	32.4 (34.03)
Rectus femoris	177.75 (104.38)	181.75 (58.11)	131.44 (56.92)
Biceps femoris long head	56.05 (13.56)	56.78 (16.91)	41.58 (26.01)
Semitendinosus	57.5 (45.2)	43.4 (52.18)	26.26 (15.78)
Gluteus maximus	41.95 (77.23)	42.85 (81.31)	26.6 (26.29)
Multifidus	140.25 (49.88)	145.75 (56.25)	100.93 (25.5)
Internal oblique	31.6 (35.61)	29.65 (34.98)	17.03 (18.18)

*BF* back-to-front, *FB* front-to-back; *CIS* conventional isometric squat.

**Table 3:** The results of statistical analysis.

Muscles	Comparison between variables	One-way ANOVA F-value or Kruskal–Wallis χ^2^	One-way ANOVA or Kruskal–Wallis p-value	Post hoc p-value *p < 0.05^a^	Cohen's d (95% confidence interval)
Abductor hallucis	BF vs. FB	11.311	*0.003	0.832	0.335 (− 0.482 to 1.128)
	BF vs. CIS			*0.006	1.400 (0.465 to 2.239)
	FB vs. CIS			*0.022	1.180 (0.277 to 2.002)
Tibialis anterior	BF vs. FB	14.710	*< 0.001	0.280	− 0.957 (− 1.765 to − 0.082)
	BF vs. CIS			0.270	1.432 (0.492 to 2.274)
	FB vs. CIS			*< 0.001	2.143 (1.077 to 3.060)
Tibialis posterior	BF vs. FB	16.114	*< 0.001	0.768	0.522 (− 0.308 to 1.317)
	BF vs. CIS			*< 0.001	1.419 (0.481 to 2.259)
	FB vs. CIS			*0.005	1.535 (0.579 to 2.386)
Peroneus longus	BF vs. FB	19.182	*< 0.001	0.239	0.589 (− 0.247 to 1.385)
	BF vs. CIS			*< 0.001	2.390 (1.275 to 3.341)
	FB vs. CIS			*0.002	1.828 (0.822 to 2.708)
Rectus femoris	BF vs. FB	0.497	0.780	0.768	− 0.229 (− 1.024 to 0.582)
	BF vs. CIS			1.000	− 0.043 (− 0.842 to 0.758)
	FB vs. CIS			0.862	0.218 (− 0.592 to 1.013)
Biceps femoris long head	BF vs. FB	1.617	0.214	0.998	− 0.023 (− 0.822 to 0.778)
	BF vs. CIS			0.291	0.631 (− 0.209 to 1.428)
	FB vs. CIS			0.226	0.674 (− 0.170 to 1.472)
Semitendinosus	BF vs. FB	6.731	*0.004	0.666	0.302 (− 0.513 to 1.096)
	BF vs. CIS			*0.004	1.663 (0.685 to 2.526)
	FB vs. CIS			*0.032	1.153 (0.253 to 1.973)
Gluteus maximus	BF vs. FB	5.407	0.067	0.955	0.009 (− 0.791 to 0.809)
	BF vs. CIS			0.107	0.991 (0.112 to 1.801)
	FB vs. CIS			0.121	0.963 (0.088 to 1.772)
Multifidus	BF vs. FB	6.731	*0.004	0.999	0.016 (− 0.784 to 0.816)
	BF vs. CIS			*0.009	1.425 (0.486 to 2.266)
	FB vs. CIS			*0.010	1.377 (0.446 to 2.214)
Internal oblique	BF vs. FB	4.092	0.129	0.955	− 0.046 (− 0.845 to 0.756)
	BF vs. CIS			0.193	0.606 (− 0.232 to 1.403)
	FB vs. CIS			0.193	0.614 (− 0.225 to 1.410)

*FB* front to back, *BF* back to front, *CIS* conventional isometric squat.

^a^Adjusted p-value by multiplying the original p-value by three.

## Discussion

The HSS demonstrated a higher activation in the Abd H, TA, TP, PL, ST, and Mul muscles compared with the CIS. The results of this study supported the hypothesis. There have been no previous reports showing biological responses to hammer swinging training, indicating the possibility of a new training method.

Perturbation-based training is effective for trunk stabilization, and it reduces pain. Schäfer et al. examined instability situations using an unstable surface or water-filled pipe or a push from a third party and stated that perturbation-based trunk stabilization training is possibly effective in improving the physical function of the lower back in elite rowers^7^. Perturbation-based intervention (using soft pads, balance cushions, BOSU balls, inverted BOSU balls, Swiss balls, slashpipes, and sling trainers) for a year in adolescent athletes reduced strength imbalances and LBP intensity decreased^8^. The Mul muscle contributes to the stability of the lumbar spine and plays a role in controlling intersegmental motion^24^. In the present study, the HSS demonstrated significant activation of the Mul muscle by perturbation-based intervention.

The foot is a complex structure that plays an essential role in maintaining static and dynamic posture. Intrinsic and extrinsic muscles control the movement and stability of the foot arch^25^. Because HSS requires the center of pressure to shift forward and backward, the TP, TA, and PL, which are fundamental muscles to control the foot arch dynamically, increased the muscle activities with HSS. Exercise programs improve intrinsic and extrinsic foot muscles to help sports injuries^26^, rehabilitation^27,28^, and prevent fall risks^29^. Short-foot exercise is a proper strengthening exercise to activate the foot muscles, especially the Abd H^27,30^, and can help strengthen the Abd H muscle in individuals with pes planus^31^. Kulig et al. have reported that TP training with orthoses could improve foot functional index scores including pain and disability^27^. Selective training for the TP with iliopsoas stretching demonstrated prominent improvements in dynamic balance and static arch height compared with conventional towel curl exercises in participants with pronated feet^30^. Recreational runners who performed a foot exercise program had a 2.42-fold lower risk of running-related injuries compared to the control group^26^. Further, 6 weeks of short-foot exercise intervention reduced navicular drop, foot pronation, foot pain, disability, and increment in plantar force of the medial midfoot in pes planus^32^. These previous studies indicate the importance of improving the function of the foot and ankle muscles.

The results of this study showed that the activity of foot and ankle muscles was increased in HSS that required postural control in the weight-bearing position. In unstable conditions, the activities of flexor digitorum longus, peroneus brevis and TA were increased^33^. The activities of the abductor pollicis brevis, flexor digitorum brevis, and plantaris quadratus muscles were analyzed in different positions, and these muscles were activated more during weight-bearing^34^. The activity of the PL and abductor pollicis longus muscles during the short-foot exercise was significantly higher in the standing position than in the sitting position^31,35^. Therefore, the present study supported the results of previous studies regarding the point that adding postural control and instability elements to exercises increases the activity of foot muscles.

In the present study, HSS significantly activated intrinsic and extrinsic foot muscles without the intention of moving the foot fingers by hammer perturbation.

The present study added an unstable situation with hammer perturbation during a squat called the HSS. In postural stability against perturbation, the activity of muscle groups related to the lumbar spine, foot, and ankle joint tended to increase rather than the muscle groups associated with the hip joint and pelvis stability. The foot and Mul muscles showed significantly higher activation without changing positions, trying to move the hip and knee joint angles or foot toes. Although this exercise shows muscle activity at BF and FB phases differently, it requires a switching function on muscle activation while swinging the hammer steadily in the squat position. Learning the timing of on and off for the muscle activation, and this movement may potentially improve the coordination of the muscles around the trunk, hip joints, knee joints, and ankle joints and help develop coordination of the whole body.

Based on the results of this study, the HSS exercise could be effective as a core and foot stability exercise. To increase the activity of the ankle muscles, toe exercises such as towel gathering and short-foot exercises should be performed. However, there are a certain number of patients who have difficulty performing these exercises due to deformity or pain. The HSS used in this study may increase the activity of the foot and ankle muscles unconsciously without consciously performing foot and ankle joint exercises. In this study, hammers were attached to the bar. If a hammer is not available in the training environment, other weights such as kettlebells, water bottles, or sandbags could be used alternatively.

This study has some limitations. First, we only examined participants in a single body position, while in the isometric squat position. Different body positions can lead to different muscle activation. In addition, the joint angles of the trunk and lower extremities during the trial were not analyzed. However, the knee angle was defined before the trial and the examiner confirmed that the posture did not change during the trial. Second, we only compared the anteroposterior direction of the hammer movement. Different hammer movements can result in different muscle activation outcomes. Third, the order of exercises in this study was not randomized. Moreover, the outcome of this study was only the amount of muscle activity, which indicates the biological response during the exercises, and the effects during the exercises were not verified. Lastly, we did not normalize the EMG signals because data were collected/compared in the same participant during the same session within a short period^23^. Therefore, these factors should be considered and analyzed in future studies. Further research is also needed to analyze not only muscle activity but also balance and other performance variables to verify the mid- to long-term effectiveness of HSS.

## Conclusion

The TA, TP, Abd H, and PL muscles were significantly more activated during HSS compared with CIS. The HSS exercise can be considered for trunk and foot stability exercise.

## Data Availability

The datasets used and/or analysed during the current study available from the corresponding author on reasonable request.
